# Replication study identified *EFEMP1* association with varicose vein predisposition among Indians

**DOI:** 10.1186/s40001-024-01786-8

**Published:** 2024-04-12

**Authors:** Rohit Mehra, Vikram Patra, Rishi Dhillan, Anuka Sharma, Sonal Kashyap, Garima Rastogi, Love Gupta, Reena Singh, Chirag Chopra, Varun Sharma

**Affiliations:** 1 Department of Vascular and Endovascular Surgery, Command Hospital (Southern Command), Pune, India; 2 Department of Vascular and Endovascular Surgery, Command Hospital (Northern Command), Udhampur, India; 3 Department of Vascular and Endovascular Surgery, Army Hospital (Research and Referral), Delhi, India; 4 NMC Genetics India Pvt. Ltd. Gurugram, Haryana, 122001 India; 5 School of Bioengineering and Biosciences, Lovely Professional University, Phagwara, Punjab India

**Keywords:** *EFEMP1*, Varicose veins, Genotyping, Indians

## Abstract

**Background:**

Varicose vein is a chronic condition that affects the lower extremities of the human body. Several factors have been implicated in the development of this disease, viz age, gender, weight, height and prolonged standing. Recently, genome-wide studies have identified genetic biomarkers that are associated with varicose veins in different ethnic groups. Such genetic studies are lacking in South Asians specifically in Indians where the prevalence of varicose veins is high, and it is important to replicate these variants in the stated population. The study aimed to replicate the association of genetic variants associated with varicose veins in this target population, which were found to be associated with the other ethnic groups.

**Methodology:**

The studied cohort is of the Indian population comprising unrelated 104 varicose veins cases and 448 non-varicose vein controls. The samples were genotyped using the Illumina Global Screening Array. Using the genomic data from UK BioBank and 23andMe studied cohorts; eight genetic variants were selected to replicate in our dataset. The allelic association was performed to identify the effective allele and risk was estimated using odds ratio and *p*-value as level of significance. Multifactor Dimensionality Reduction was used to estimate the cumulative effect of variants in Indians.

**Result:**

Variant rs3791679 of *EFEMP1* was found to be associated with varicose veins in Indians. After observing the association of the *EFEMP*1 with varicose veins, we further ensued to identify all genetic variants within *EFEMP1* to uncover the additional variants associated with this trait. Interestingly, we identified six new variants of EFEMP1 gene that have shown association. Moreover, the cumulative effect of all associated variations was estimated and the risk was 2.7 times higher in cases than controls whereas independently their effect ranges from 0.37–1.58.

**Conclusion:**

This study identifies *EFEMP1* as a potential gene related to the risk of varicose veins in Indians. It also highlights that evaluating the maximum number of variants of a gene rather than focusing solely on replicating single variations offers a more comprehensive and nuanced understanding of the genetic factors contributing to a complex trait like varicose veins.

**Supplementary Information:**

The online version contains supplementary material available at 10.1186/s40001-024-01786-8.

## Introduction

Varicose veins (VV) is a prevalent venous disorder characterized by the dilated, tortuous, elongated subcutaneous veins of the legs that occur due to a multitude of factors, primarily affecting either the vein valve functionality or the vein wall [1]. To obtain a degree of uniformity one of the most common classifications used for VV is CEAP classification (clinical, etiological, anatomical, and pathophysiological) [2, 3]. Clinically, the patients present with one of the following: complaints, cosmesis or complications of varicose veins, with venostatic symptoms being the most common presentation, closely followed by the need for esthetics [4].

Characteristic features of VV include dilated, tortuous, elongated subcutaneous veins that appear on the lower extremities. It causes discomfort, pain, and cosmetic concerns, and in some cases, leads to severe morbidity [5]. The pathophysiology of VV involves a complex interplay of venous insufficiency and valvular dysfunctions [6]. Several theories have been forecasted and tested in the last century including the leucocyte entrapment theory, fibrin cuff theory and the ambulatory venous hypertension theory. Despite all odds ambulatory venous hypertension theory has held its ground and is the most accepted theory in the contemporary world [7]. Collaborative evidence directs towards the valvular dysfunction which primarily affects the one-way valves in the veins, contributing to the retrograde flow of blood and further exacerbating venous insufficiency [8]. These collectively result in involvement of the vein valve and the vein wall thus leading to venous dilation, increased wall tension, and subsequent varicose vein formation [9, [10].

The reported prevalence of VV varies from 5 to 60% depending on the age group, underscoring the urgent need to identify the contributing variables [11–14]. Numerous risk factors have been identified that contribute to the development and progression of varicose veins. Advancing age, genetic predisposition, and female gender are considered non-modifiable risk factors [15]. Hormonal influences, such as pregnancy and hormonal replacement therapy are implicated in worsening the condition [16]. Lifestyle factors, including occupations demanding prolonged standing, obesity, and lack of physical activity, are modifiable risk factors that can be addressed to reduce the likelihood of varicose vein [17]. Review of the extant world literature reveals that different ethnicities have different disease-related genes that induce VV [11, 18–21]. Genetic studies of VV in Indians are almost absent and only CASZ1 variants have shown susceptibility towards the risk of VV in South Asian Indians by our research group (unpublished study). To identify more genetic variants associated with VV to explain the missing heritability in the background of significant disease burden of VV in Indians, the present study has replicated the newly identified risk loci in South Asian Indians. This study was an endeavor to provide insight into the role of genetics in the development of VV and to decipher potential risk markers in the studied population group.

## Materials and methods

### Sample collection criteria

The study cohort of 552 individuals comprised 104 cases of primary VV and 448 controls. The clinical, etiological, anatomical, and pathological (CEAP) classification was used to categorize these individuals [22]. Each patient was assessed by a group of expert vascular surgeons. A phlebotomist collected 3–4 mL of blood under stringent aseptic conditions after obtaining the consent of the patient in ethylenediamine tetra acetic acid (EDTA) vials which were maintained at 4 °C. The study was approved by Institutional Ethical committee (IEC reg No. 35/2021).

### DNA isolation and genotyping

DNA was isolated using the QIAGEN DNA Mini Kit by following the manufacturer’s protocol. The quality and concentration of DNA was checked on 1% Agarose gel. After screening of the DNA samples, they underwent the genotyping using the Illumina Global Screening Array. The genotyping process was adapted from our earlier work [23]. The genotype call rate observed was greater than 98% and GenomeStudio 2.0 software was used to convert the genotyping data into plink format files for further analysis.

### SNP selection

Eight genetic variations that were found to be associated with VV in UK Bio Bank and 23andMe cohort were taken for the evaluation [24] and were also present in our dataset. The details of the replicated variants are summarized in Additional file 1: Table S1.

### Data analysis

Continuous socio-demographic, anthropometric and clinical data were provided as an average and count of individuals, with frequency expressed as a percentage and standard deviation (Additional file 1: Table S2).The genetic data analysis was done using the plink 1.09 [25]. Hardy–Weinberg equilibrium (HWE) was determined for each variation. Variant with call rate of < 95% and minor allele frequency < 0.05, or deviation from HWE (*P*_hwe_ < 0.05) were excluded from downstream analysis. After data quality check, the genotypes of each variant were extracted using command—*extract snps.txt—recode* from the large dataset. The association of variants was estimated using odds ratio and 95% confidence interval (CI) with respect to minor allele frequency. The synergic effect of significantly associated SNPs was estimated using Multifactor Dimensionality Reduction (MDR) software [26]. The variations that were found to be associated were further evaluated for the linkage disequilibrium using Haploview [27].

## Results

The replication study included 104 patients with primary VV and 448 control samples. The socio-demographic details of cases and controls are summarized in Additional file 1: Table S2. The present cohort of VV was categorized according to the CEAP classification system and it showed the following distribution pattern: 7.77% of cases fell under C1disease; the majority, accounting for 53.40% of cases, were classified as C2 disease, signifying the presence of varicose veins; 14.56% were categorized as C3, indicating edema; while 10.68%, 8.74%, and 4.85% were classified as C4 (pigmentation and/or eczema), C5 (healed ulcer), and C6 (active ulcer), respectively. These findings are summarized in Additional file 1: Table S3.

Initially, the association was carried out among eight genetic variants which were previously found to be associated with VV in GWAS (Additional file 1: Table S1). Out of these eight genetic variants, *EFEMP1* variant rs3791679 was found to be significantly associated with VV in the studied cohort. The OR observed for *EFEMP1* variant rs3791679 was 1.43 (1.04–1.97, at 95% CI) implicating it as a potential gene causing risk of VV in Indians (Table 1).

**Table 1:** Replication of GWAS variants in Indians

Chr	SNP	Gene	Chromosome location (GRCh37)	OR	L95	U95	*p*-value
1	rs2820464	*LYPLAL1-AS1*	1:219,693,220	1.19	0.85	1.66	0.31
2	rs3791679*	*EFEMP1*	2:56,096,892	1.43	1.04	1.97	0.02
3	rs2713575	*LINC01565 / GATA2*	3:128,294,355	0.92	0.68	1.25	0.59
4	rs11728719	*SORBS2*	4:186,696,172	1.10	0.72	1.68	0.65
6	rs1936800	*LOC105377989 / RSPO3*	6:127,436,064	0.98	0.72	1.32	0.88
8	rs10504825	*CPNE3*	8:87,567,848	0.85	0.62	1.18	0.34
10	rs61863928	*-*	10:64,449,549	0.98	0.72	1.34	0.88
16	rs11076178	*CPNE2*	16:57,146,402	0.89	0.61	1.31	0.56

*Chr* chromosome, *SNP* single nucleotide polymorphism, *OR* odds ratio, *L95 and U95* 95% lower and upper confidence interval

rs3791679* of EFEMP1 gene showed association

**Fig. 1:**
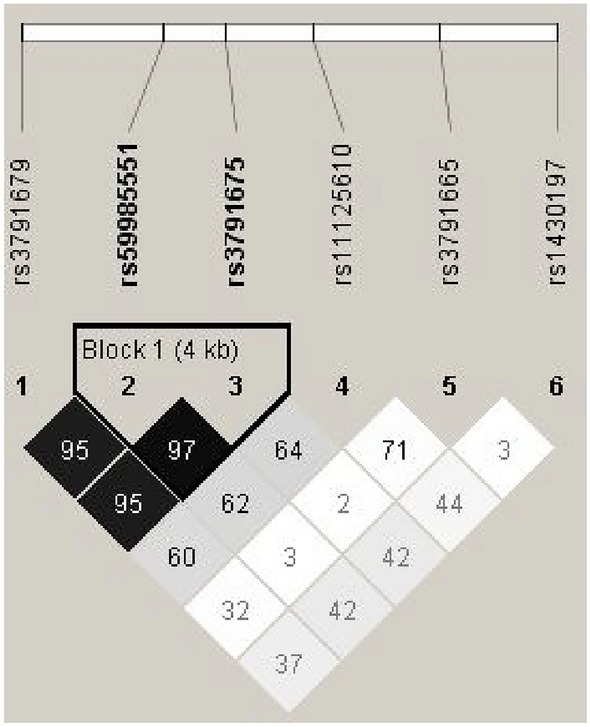
Linkage disequilibrium observed among the six associated SNPs of *EFEMP1* in Indians. rs59985551 and rs3791675 of *EFEMP1 *were observed to be in strong LD with *r*^2^0.97

Considering *EFEMP1* as a potential candidate gene for VV in Indians, we evaluated all the variations of *EFEMP1* present in our dataset. A total of 27 variants were screened. After applying quality check parameters, 21 variations (Additional file 1: Table S4) passed the quality check. Out of these 21 variations, six variants were found to be associated on the basis of their OR and *p*-value. The OR had a range of 0.37–1.58 and *p*-value of < 0.05 (Table 2). The variant rs3791665 which was causing protection had an OR of 0.37 and the other five variations rs3791679, rs59985551, rs3791675, rs11125610, rs1430197 were causing risk of VV and had a high OR of 1.36–1.58 (Table 2). Furthermore, in the evaluation of linkage disequilibrium (LD) among the six associated SNPs of *EFEMP1*, a robust LD association was observed between rs59985551 and rs3791675 at a genomic distance of 4 kb, with an *R*^2^ 0.94 (Fig. 1) in the studied cohort.

**Table 2:** Effective allele frequency distribution and *EFEMP1* variant showing association with VV in South Asian Indians

CHR	SNP	BP	OR	L95	U95	*p*-value	CES OR
2	rs3791679	56,096,892	1.43	1.04	1.97	0.03	2.73 (1.75–4.23)
2	rs59985551	56,106,928	1.42	1.03	1.97	0.03	
2	rs3791675	56,111,309	1.42	1.03	1.96	0.03	
2	rs11125610	56,117,614	1.58	1.05	2.37	0.03	*p*-value < 0.0001
2	rs3791665	56,126,509	0.37	0.16	0.86	0.02	
2	rs1430197	56,134,827	1.36	1.00	1.83	0.05	

*CHR* chromosome, *SNP* single nucleotide polymorphism, *BP* base pairs, *EA* effective allele, *EAF* effective allele frequency, *CES* cumulative effect of SNPs calculated using MDR with a balanced accuracy of > 0.75 and specificity and sensitivity of > 0.80, OR Odds ratio

The interactive association of LD variants was also estimated and no association was observed OR 1.50 (95%CI 0.80–2.81, *p*-value 0.20). This observation prompted us to explore the cumulative effect of all six SNPs in the studied cohort to shed light on the potential synergistic impact of these genetic variations on VV susceptibility. Assessment of the cumulative effect of six significantly associated variants revealed that the risk increases 2.7 times of the risk caused by independent variants. The OR observed was 2.73 (1.75–4.23, 95% CI) with a *p*-value < 0.00001. MDR analysis showed that the risk contribution of all six associated variants increased to 2.7 times than the independent risk, indicating multiple small effects of different variants of EFEMP1 gene plays crucial role in the development of VV in the studied population group.

## Discussion

Varicose veins are defined as dilated, tortuous, elongated subcutaneous veins of ≥ 4 mm diameter. A multitude of risk factors like chronic cough, constipation, family history of venous diseases, gender, obesity, advanced age, pregnancy, and prolonged periods of standing have been implicated in the development of VV. Although precise pathophysiology is a subject of debate, VV is believed to encompass a genetic predisposition leading to dysfunctional vein valves, compromised vascular walls, expressing as elevated venous pressure within the axial veins and tributaries [28]. The heredity seems to influence the etiology, yet the genetic factors are poorly understood. Decoding this elusive genetic component will open new frontiers to determine the molecular triggers of the disease, extending insights into the pathophysiology, and redefine the goals of management.

Several studies have been conducted to find out the genetic association of different genes with VV [18, 19, 29–32]. In this study, we found that *EFEMP1* gene and its variants play an important role in the pathophysiology of VV. The gene shows significant association in the studied Indian cohort and it emphasizes its genetic role in the development of VV, as already proven in the German population [33].

Previously, we found out that *CASZ1* gene has shown susceptibility towards association with VV (paper under revision) in South Asian Indians. Taking lead, we did a replication study for another eight genetic variants which have been studied in Europeans [24]. Out of these variations, one of the variations rs3791679 of *EFEMP1* gene showed a strong association as a risk factor for VV in Indian cohort.

As we investigated the other 27 variations of *EFEMP1* gene and screened them for their potential link to varicose veins, only six variations passed quality check parameters. These 6 variations were responsible for the development of VV and had OR of 0.37–1.58 and a *p*-value of < 0.05 and were analyzed using reactome pathway database [34] (Additional file 1: Fig. S1). These variants were found to play a significant role in pathways linked to extracellular matrix organization and molecules associated with elastic fibers thus indicating association with maintenance of structural and mechanical integrity of the vein’s architecture. They thus hold the potential to serve as genetic markers for detecting VV in individuals of Indian descent.

Open Targets Genetics platform [35] was used to confirm the association between six variants of *EFEMP1* using a phenome-wide association study (PheWAS) approach) using GWAS catalog and UK BioBank database. One of the lead variant (rs3791679) demonstrated an association with various phenotypes including VV (Additional file 1: Fig. S2). Five variations were found to offer at risk potential and one variation offered protection.

To ascertain the cumulative effect of these 6 preferably use one connotation only, either numerical 6 or alphabet six variants, MDR was performed for the associated SNPs. We found that their contribution towards the risk of VV increased 2.7 times as compared to their individual risks. It shows that the multiple small effects of different variants of *EFEMP1* gene plays crucial role in the development of VV in the studied population group.

The present study provides an insight into studying multiple variants within a single gene for a complex trait and necessity of extending our findings to larger and more diverse ethnic cohorts. To establish *EFEMP1* as a viable candidate gene for VV additional research involving different ethnicities to investigate the variations linked to the disease risk.

The present study heralds a conscious reasoning into the complex genetic landscape of VV, stressing the importance of EFEMP1 gene variations in the Indian population. The outcomes echo existence of potential genetic markers for detecting VV and highlight the role of numerous genetic variations of same phenotypic disease development. This study contributes to a better scientific understanding of the genetic basis of VV in an unexplored population cohort with a sizable disease burden. The findings might have far reaching ramifications in shaping management protocols with advent of further translational research.

### Supplementary Information

**Additional file 1: Table S1.** Genetic Variants that were replicated in Indians and are adopted from GWAS. **Table S2.** Socio Demographic details observed in Case and Control. **Table S3.** Frequency of CEAP classification observed in the studied cases of varicose veins. **Table S4.** Twenty one variants of EFEMP1 that have passed the QC and evaluated for association of VV in studied cohort. **Table S5.** Allele frequency and functional annotation of EFEMp1 variants observed in the 1000genome and gnomAD. **Figure S1.** Pathway analysis of EFEMP1 variants using Reactome pathway database. **Figure S2.** The forest plot depicting various traits associated with the lead variant of EFEMP1 (rs3791679) revealed significant connections with different phenotypes, including deep vein thrombosis, chronic venous insufficiency, etc. https://genetics.opentargets.org/ was used to access the data of GWAS catalog and UK biobank.

## Data Availability

The datasets utilized and/or analyzed during the present study are accessible from the corresponding author upon reasonable request.
