# Supplementary material for: Replication study identified EFEMP1 association with varicose vein predisposition among Indians
Source: Eur J Med Res. 2024 Apr 12;29:232. doi: 10.1186/s40001-024-01786-8 (PMC11015598; doi:10.1186/s40001-024-01786-8)
Supplement: Supplementary file 1 — Additional file 1: Table S1. Genetic Variants that were replicated in Indians and are adopted from GWAS. Table S2. Socio Demographic details observed in Case and Control. Table S3. Frequency of CEAP classification observed in the studied cases of varicose veins. Table S4. Twenty one variants of EFEMP1 that have passed the QC and evaluated for association of VV in studied cohort. Table S5. Allele frequency and functional annotation of EFEMp1 variants observed in the 1000genome and gnomAD. Figure S1. Pathway analysis of EFEMP1 variants using Reactome pathway database. Figure S2. The forest plot depicting various traits associated with the lead variant of EFEMP1 (rs3791679) revealed significant connections with different phenotypes, including deep vein thrombosis, chronic venous insufficiency, etc. https://genetics.opentargets.org/ was used to access the data of GWAS catalog and UK biobank. [file 40001_2024_1786_MOESM1_ESM.docx]

Table S1: Genetic Variants that were replicated in Indians and are adopted from GWAS (Ahmed et al., 2022)

| Chr | SNP | Position | EA | Candidate Gene | EAF in UK bio bank cohort | EAF in 23 and me cohort |
| --- | --- | --- | --- | --- | --- | --- |
| 1 | rs2820464 | 219693220 | A | *-* | 0.34 | 0.32 |
| 2 | rs3791679 | 56096892 | A | *EFEMP1* | 0.77 | 0.76 |
| 3 | rs2713575 | 128294355 | G | *GATA2* | 0.5 | 0.5 |
| 4 | rs11728719 | 186696172 | A | *SORBS2* | 0.76 | 0.77 |
| 6 | rs1936800 | 127436064 | C | *RSPO3* | 0.47 | 0.49 |
| 8 | rs10504825 | 87567848 | C | *CPNE3* | 0.41 | 0.41 |
| 10 | rs61863928 | 64449549 | G | *-* | 0.62 | 0.64 |
| 16 | rs11076178 | 57146402 | T | *CPNE2* | 0.11 | 0.11 |

Chr = chromosome, SNP = Single nucleotide polymorphism, EA = Effective Allele, EAF = Effective allele Frequency

Table S2: Socio Demographic details observed in Case and Control

| Clinical Characteristics | Cases  (n= 104) | Controls  (n= 448) |
| --- | --- | --- |
| Age  in years (± SD) | 47.5 ± 17 | 52.3 ± 15 |
| Weight  In kilograms(±SD) | 71.16 ± 14 | 68.1 ± 14 |
| Height in centimeters(±SD) | 168.08 ± 8.95 | 168.6 ± 10 |
| Gender  (in percentage) | F=32.04  M=67.96 | F=34.61  M=65.39 |
| Smoking  (in percentage) | Yes= 27.18,  No= 72.82 |  |
| Standing occupation  (in percentage) | Yes= 59.22,  No= 40.78 |  |
| Prolonged sitting  (in percentage) | Yes= 13.59,  No= 86.41 |  |
| Deep Vein Thrombosis  (in percentage) | Yes= 11.65,  No= 88.35 |  |

Table S3: Frequency of CEAP classification observed in the studied cases of varicose veins

| CEAP Classification  N= 104 | Frequency  in Percentage |
| --- | --- |
| C1 | 7.77 |
| C2 | 53.40 |
| C3 | 14.56 |
| C4 | 10.68 |
| C5 | 8.74 |
| C6 | 4.85 |

C1= Telangiectasies or reticular veins, C2 Varicose veins; distinguished from reticular veins by a diameter of 3mm or more, C3 Edema, C4 Changes in skin and subcutaneous tissue secondary to CVD, C5 Healed venous ulcer, C6 Active venous ulcer (Lurie et al., 2020)

Table S4: Twenty one variants of *EFEMP1* that have passed the QC and evaluated for association of VV in studied cohort

| Chr | SNP | EA | EAF in cases | OR | L95 | U95 | p- value |
| --- | --- | --- | --- | --- | --- | --- | --- |
| 2 | **rs3791679** | **G** | **0.351** | **1.428** | **1.036** | **1.967** | **0.03** |
| 2 | rs7563085 | A | 0.06311 | 0.5816 | 0.3188 | 1.061 | 0.07 |
| 2 | rs72807772 | A | 0.2356 | 1.138 | 0.7953 | 1.627 | 0.48 |
| 2 | **rs59985551** | **A** | **0.3465** | **1.424** | **1.029** | **1.972** | **0.03** |
| 2 | rs1346786 | A | 0.3894 | 1.2 | 0.8792 | 1.637 | 0.25 |
| 2 | rs11680866 | A | 0.2981 | 1.039 | 0.7466 | 1.445 | 0.82 |
| 2 | rs3791676 | A | 0.2816 | 0.8712 | 0.6232 | 1.218 | 0.42 |
| 2 | **rs3791675** | **A** | **0.3431** | **1.42** | **1.026** | **1.964** | **0.03** |
| 2 | rs1367228 | C | 0.3125 | 0.8274 | 0.5986 | 1.144 | 0.25 |
| 2 | rs11125609 | G | 0.4038 | 1.346 | 0.9873 | 1.834 | 0.06 |
| 2 | rs1430192 | A | 0.2644 | 0.725 | 0.517 | 1.017 | 0.06 |
| 2 | **rs11125610** | **G** | **0.1796** | **1.577** | **1.048** | **2.372** | **0.03** |
| 2 | rs1430193 | T | 0.5337 | 1.309 | 0.9671 | 1.771 | 0.08 |
| 2 | **rs3791665** | **A** | **0.02885** | **0.3657** | **0.1564** | **0.8551** | **0.02** |
| 2 | rs3791664 | G | 0.3173 | 1.259 | 0.9079 | 1.747 | 0.17 |
| 2 | rs1430195 | G | 0.2933 | 0.9903 | 0.711 | 1.379 | 0.95 |
| 2 | **rs1430197** | **A** | **0.5288** | **1.355** | **1.001** | **1.833** | **0.05** |
| 2 | rs75046370 | G | 0.009615 | 1.437 | 0.2879 | 7.17 | 0.66 |
| 2 | rs14282 | G | 0.1106 | 1.563 | 0.9477 | 2.579 | 0.08 |
| 2 | rs2903838 | G | 0.5144 | 1.103 | 0.8155 | 1.492 | 0.52 |
| 2 | rs79563212 | A | 0.02885 | 1.301 | 0.5159 | 3.281 | 0.58 |

Chr = chromosome, EA = Effective allele, EAF = Effective Allele Frequency in cases, OR = Odds ratio, L95 = Lower confidence interval, U95 = Upper confidence interval, variants in bold showed association with VV in studies cohort

Table S5: Allele frequency and functional annotation of EFEMp1 variants observed in the 1000genome and gnomAD

| SNPs | Chr:location | Allele | Consequence | Gene | 1000G | gnomAD | CADD_PHRED | CADD_RAW |
| --- | --- | --- | --- | --- | --- | --- | --- | --- |
| rs3791679 | 2:55869757-55869757 | T | intron_variant | EFEMP1 | - | - | 19.47 | 2.028058 |
| rs7563085 | 2:55876035-55876035 | A | intron_variant | EFEMP1 | 0.122 | 0.126 | 0.159 | -0.439727 |
| rs72807772 | 2:55877176-55877176 | A | intron_variant | EFEMP1 | 0.0751 | 0.04856 | 16.91 | 1.681795 |
| rs59985551 | 2:55879793-55879793 | T | intron_variant | EFEMP1 | 0.3005 | 0.2197 | 5.908 | 0.443895 |
| rs1346786 | 2:55881198-55881198 | T | intron_variant | EFEMP1 | 0.4569 | 0.3678 | 2.167 | 0.108333 |
| rs11680866 | 2:55881554-55881554 | A | intron_variant | EFEMP1 | 0.1436 | 0.1303 | 3.121 | 0.199787 |
| rs3791676 | 2:55883417-55883417 | A | intron_variant | EFEMP1 | - | - | 16.86 | 1.674798 |
| rs3791675 | 2:55884174-55884174 | T | intron_variant | EFEMP1 | 0.2905 | 0.2151 | 1.083 | -0.04408 |
| rs1367228 | 2:55885305-55885305 | A | intron_variant | EFEMP1 | 0.6556 | 0.5435 | 2.98 | 0.187334 |
| rs11125609 | 2:55888699-55888699 | C | intron_variant | EFEMP1 | 0.3462 | 0.2942 | 1.678 | 0.049746 |
| rs1430192 | 2:55889905-55889905 | C | intron_variant | EFEMP1 | 0.7394 | 0.642 | 4.691 | 0.334824 |
| rs11125610 | 2:55890479-55890479 | C | intron_variant | EFEMP1 | 0.1733 | 0.161 | 1.555 | 0.032921 |
| rs1430193 | 2:55893718-55893718 | T | intron_variant | EFEMP1 | 0.6138 | 0.4869 | 0.908 | -0.080925 |
| rs3791665 | 2:55899374-55899374 | T | intron_variant | EFEMP1 | 0.1054 | 0.06915 | 1.473 | 0.021084 |
| rs3791664 | 2:55899956-55899956 | C | intron_variant | EFEMP1 | 0.2875 | 0.2376 | 19.07 | 1.975927 |
| rs1430195 | 2:55903505-55903505 | G | intron_variant | EFEMP1 | 0.4121 | 0.3846 | 4.022 | 0.27708 |
| rs1430197 | 2:55907692-55907692 | C | intron_variant | EFEMP1 | - | 6.58E-06 | 0.134 | -0.474718 |
| rs75046370 | 2:55910026-55910026 | C | intron_variant | EFEMP1 | 0.0088 | 0.001997 | 0.295 | -0.311146 |
| rs14282 | 2:55917795-55917795 | C | synonymous_variant | EFEMP1 | 0.0194 | 0.02028 | 12.08 | 1.037025 |
| rs2903838 | 2:55923267-55923267 | G | intron_variant | EFEMP1 | 0.4113 | 0.3825 | 0.366 | -0.266985 |
| rs79563212 | 2:55924122-55924122 | T | upstream_gene_variant | EFEMP1 | 0.0675 | 0.06329 | 6.892 | 0.537561 |


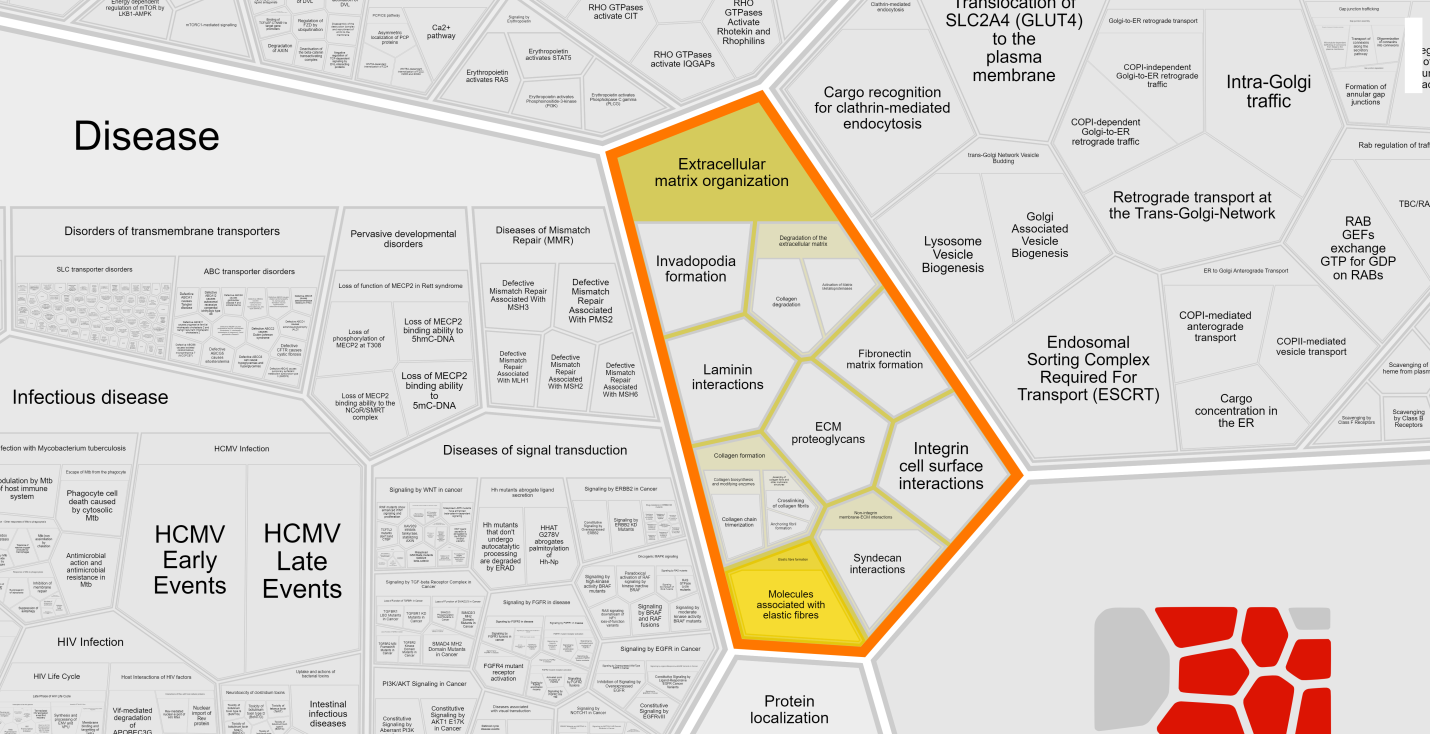


Figure S1: Pathway analysis of *EFEMP1* variants using Reactome pathway database (Fabregat et al., 2017).

Six variants that have demonstrated positive association were analyzed using the Reactome pathway database. These variants have been found to play a significant role in pathways linked to extracellular matrix organization and molecules associated with elastic fibers indicating that the variants play an important role in maintaining the structural and mechanical integrity of the veins. These variants hold the potential to serve as genetic markers for detecting varicose veins in individuals of Indian descent.


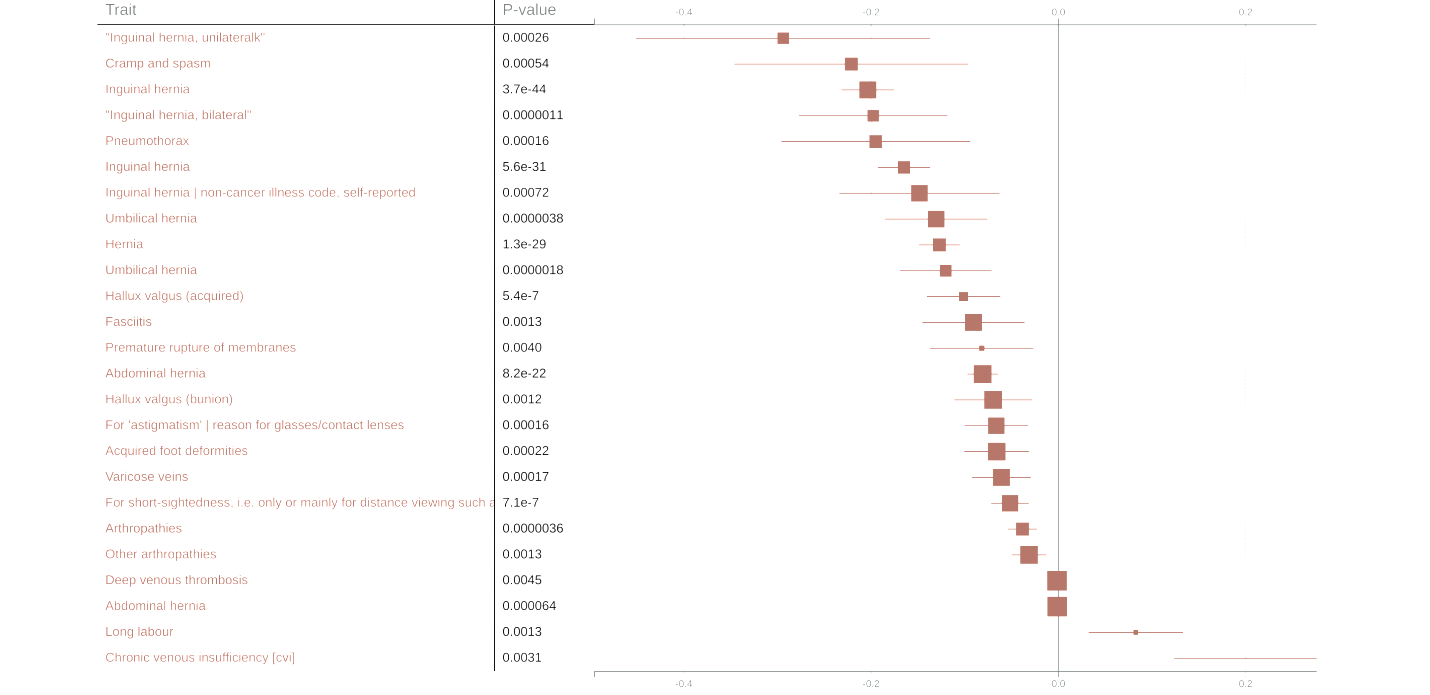


Figure S2: The forest plot depicting various traits associated with the lead variant of *EFEMP1* (rs3791679) revealed significant connections with different phenotypes, including deep vein thrombosis, chronic venous insufficiency, *etc*. <https://genetics.opentargets.org/> was used to access the data of GWAS catalog and UK biobank.

**References:**

Ahmed, W.U., Kleeman, S., Ng, M., Wang, W., Auton, A., andMe Research, T., Lee, R., Handa, A., Zondervan, K.T., Wiberg, A. and Furniss, D., 2022. Genome-wide association analysis and replication in 810,625 individuals with varicose veins. Nat Commun 13, 3065.

Fabregat, A., Sidiropoulos, K., Viteri, G., Forner, O., Marin-Garcia, P., Arnau, V., D'Eustachio, P., Stein, L. and Hermjakob, H., 2017. Reactome pathway analysis: a high-performance in-memory approach. BMC Bioinformatics 18, 142.

Lurie, F., Passman, M., Meisner, M., Dalsing, M., Masuda, E., Welch, H., Bush, R.L., Blebea, J., Carpentier, P.H., De Maeseneer, M., Gasparis, A., Labropoulos, N., Marston, W.A., Rafetto, J., Santiago, F., Shortell, C., Uhl, J.F., Urbanek, T., van Rij, A., Eklof, B., Gloviczki, P., Kistner, R., Lawrence, P., Moneta, G., Padberg, F., Perrin, M. and Wakefield, T., 2020. The 2020 update of the CEAP classification system and reporting standards. J Vasc Surg Venous Lymphat Disord 8, 342-352.
